# Clinical tool to measure fluorescein patterns in orthokeratology

**DOI:** 10.7717/peerj.14068

**Published:** 2022-09-23

**Authors:** Marina López García Rosuero, Alejandro Arranz Bombin, Roberto Romero, Roberto Hornero, Raul Martin

**Affiliations:** 1 School of Optometry, Universidad de Valladolid, Valladolid, Spain; 2 Biomedical Engineering Group, Universidad de Valladolid, Valladolid, Spain; 3 IOBA Eye Institute, Universidad de Valladolid, Valladolid, Spain

**Keywords:** Orthokeratology, Fluorescein pattern, Fit evaluation, Image processing, Myopia control

## Abstract

**Background:**

Orthokeratology (ortho-k) is an overnight clinical contact lens wear technique to correct myopia and to reduce myopia progression wearing reverse-geometry rigid gas-permeable lenses. The lens fitting procedure in clinical practice usually requires subjective assessment of fluorescein pattern (fluorescein “bull’s eye” pattern). The aim of this study was to develop a novel tool for fluorescein pattern measurements to reduce subjective practitioner dependency, especially in inexperienced practitioners, in ortho-k practice.

**Methods:**

A new MATLAB customized algorithm to measure the horizontal width of the four main zones of ortho-k fluorescein patterns (central bearing, tear reservoir, mid-peripheral bearing and edge lift) was designed. The algorithm was tested on a small image database consisting of 26 ortho-k fluorescein pattern images of 13 volunteers fitted with reverse geometry lenses (Seefree, Conoptica-Hecht Contactlinsen). The agreement between two independent observers and the ImageJ measurements was determined.

**Results:**

The new clinical tool provided similar measurements to ImageJ software for the central bearing (4.20 ± 0.74 and 4.27 ± 0.69 mm; *P* = 0.21), tear reservoir (1.69 ± 0.41 and 1.69 ± 0.45 mm; *P* = 0.69), mid-peripheral bearing (1.17 ± 0.11 and 1.13 ± 0.10 mm; *P* < 0.01) and edge lift (0.48 ± 0.06 and 0.48 ± 0.06 mm; *P* = 0.81) zones. Good agreement between the software (limits of agreement lower than ±0.55 mm) and inter-observer measurements (limits of agreement lower than ±0.66 mm) was found.

**Conclusions:**

The proposed clinical tool for semiautomatic fluorescein pattern measurements in ortho-k could help to reduce practitioner dependency in fluorescein pattern assessment with future potential to introduce prediction algorithms or artificial intelligence methods in myopia control management.

## Introduction

Orthokeratology (ortho-k) is an overnight clinical contact lens wear technique that is widely used worldwide to correct myopia and to reduce myopia progression in children wearing reverse-geometry rigid gas-permeable (RGP) lenses (Swarbrick, 2006; Vincent et al., 2021). Reverse-geometry RGP lens (also called ortho-k lens) design is characterized by a central optic zone fitted flat relative to the central corneal curvature, surrounded by one or more steeper curves (Swarbrick, 2006; Vincent et al., 2021). Ortho-k lenses reduce the myopic refractive error because a flatter base curve applies hydraulic pressure to the central cornea, (Alharbi & Swarbrick, 2003) inducing central epithelial thinning and mid peripheral epithelial thickening without significant stromal changes (Alharbi & Swarbrick, 2003; Nieto-Bona et al., 2011; Kim et al., 2018).

The clinical efficacy of ortho-k to reduce myopia and its progression is well established (Cho, Cheung & Edwards, 2005; Santodomingo-Rubido et al., 2012; Si et al., 2015; Huang et al., 2016; VanderVeen et al., 2019; Cho & Cheung, 2017; Vincent et al., 2021). The lens fitting procedure in clinical practice usually requires calculating lens specifications (following the manufacturer’s instructions with or without any topographic fitting software support), assessing the suitability of the lens by analyzing the fluorescein pattern with trial lenses (fluorescein “bull’s eye” pattern) and confirming a bull’s eye corneal topographic pattern (treatment zone) after overnight use of ortho-k lenses (Kim et al., 2018). This procedure can take a significant amount of time and can require several trials to determine the final lens parameters (Zhang et al., 2017). Failures of the ortho-k treatment have been related to poor lens fitting, decentered lens, or other ortho-k lens complications, such as corneal stain and keratitis (Maseedupally et al., 2016; Kam et al., 2017). The ideal fluorescein bull’s eye pattern of ortho-k lenses shows a “central bearing” area (close central optic zone alignment between the lens and cornea with a tear layer thinner than 15–20 microns (Wolffsohn, van der Worp & de Brabander, 2013)) surrounded by an annulus of fluorescein (corneal clearance under the reverse-curve zone or tear reservoir zone), followed by an area of “mid-peripheral bearing” (alignment zone under the flatter lens curve fitted in corneal alignment) and finally a narrow edge lift clearance (Swarbrick, 2006; Gasson & Morris, 2015).

Fluorescein pattern analysis is an integral part of RGP contact lens fitting (Wolffsohn, van der Worp & de Brabander, 2013) especially in ortho-k because a central alignment/bearing zone between 3.5 and 4.5 mm wide is recommended in ortho-k lens, (Mountford, Cho & Chui, 2005; Gasson & Morris, 2015) but this estimation is subjectively assessed by contact lens practitioners, using slit-lamp or other techniques. However, reliable assessment of the cornea-to-lens fitting relationship in ortho-k practice is difficult, even for experienced practitioners, so clinicians typically do not attempt to measure fluorescein pattern and simply judge whether if it is acceptable or not (Vincent et al., 2021). In fact, previous reports have found moderate agreement (40–60%) between the assessment of fluorescein patterns *via* experienced practitioners by using conventional (Wolffsohn, van der Worp & de Brabander, 2013) and aspheric (Orsborn et al., 1989) RGP lenses, but the agreement is lower than 20% with ortho-k lenses (Mountford, Cho & Chui, 2005). Thus, it is difficult to identify the optimal fitting of ortho-k lenses with only static fluorescein pattern analysis (Mountford, Cho & Chui, 2005) and currently ortho-k practice requires reliable corneal topography maps to lens selection and modifications (Vincent et al., 2021). Therefore, fluorescein analysis is highly subjective with a high practitioner dependency (Wolffsohn, van der Worp & de Brabander, 2013), and at least one night of ortho-k lens wear is compulsory to complete fitting assessment (Vincent et al., 2021). To the best of our knowledge, there is no objective system or imaging analysis tool that can quantitatively assess fluorescein patterns during ortho-k.

The purpose of this study is to develop a proof of concept of a new algorithm to conduct a semiautomatic analysis of ortho-k fluorescein patterns; this algorithm will allow measurements of the width of the four main areas of this pattern, namely, the central bearing, tear reservoir, alignment and edge lift zones along the horizontal meridian. Furthermore, the proposed algorithm can help to standardize the ortho-k fitting procedure, develop new fitting rules, simplify the co-management of patients using ortho-k, or collect enough data that could be used in future clinical and/or research practice, for example, introducing artificial intelligence to improve the prediction of an ortho-k treatment.

## Methods

### Subjects

This study included 26 eyes from 13 young adult subjects (seven men, six women) aged between 19 and 38 years (22.94 ± 4.67 years) who agreed to participate in this study. The mean spherical equivalent (sphere + ½ cylinder) ranged from −0.50 to −4.32 D (−2.24 ± 1.14 D) with refractive astigmatism lower than 1.25 D. Subjects were excluded if they had a history of ocular surgery, an active ocular surface disease and/or current medication that could affect ocular physiology and contact lens wear, and refractive or corneal astigmatism greater than 1.25 diopters. This study protocol was approved (PI-191603) by the Human Sciences Ethics Committee of Valladolid Area-Este Clinic Hospital (Castilla y Leon public health system-SACYL) and conducted according to the tenets of the Helsinki Declaration. All participants were informed about the study aim and their participation, and written informed consent to participate was obtained from each participant.

Subjects were fitted in a single visit with ortho-k lenses (Seefree; Conoptica-Hecht Contactlinsen, Barcelona, Spain), and a detailed description of the characteristics of these lenses is summarized in Table 1. The trial lens parameters were selected by a masked, independent and experienced contact lens practitioner on the basis of corneal topography (Oculus Easygraph, Wetzlar, Germany) with APEX contact lens fitting software (APEX version 1.1.0.6; Conoptica-Hecht Contactlinsen, Barcelona, Spain) to achieve an optimal fluorescein pattern with a tear layer thickness between 5 to 15 microns under the central optic zone (Mountford, Cho & Chui, 2005), giving the appearance of bull’s eye fluorescein pattern. Prior to lens insertion eye exam was conducted to assess corneal health and collect corneal topography. Just one trial lens per eye was fitted.

**Table 1:** Description of the ortho-k lenses used in the study.

Trade name	Seefree
Manufacturer	Conoptica-Hecht Contactlinsen
Total diameter (mm)	10.89 ± 0.39 (Min 10.2–Max 11.5)
BOZD (D0) (mm)	6.79 ± 0.38 (Min 6.1–Max 7.4)
Reverse-curve width (D1) (mm)	0.60
Alignment width (D2) (mm)	0.85
Edge lift width (D3) (mm)	0.60
BOZR (R0) (mm)	8.40 ± 0.39 (Min 7.65–Max 9.05)
Reverse-curve (R1) (mm)	7.19 ± 0.41 (Min 6.55–Max 8.00)
Alignment radius (R2) (mm)	8.08 ± 0.35 (Min 7.40–Max 8.70)
Peripheral curve (R3) (mm)	12.19 ± 0.01 (Min 12.0–Max 12.5)
Material	Boston XO
Nominal Dk*	100 × 10^−11^
Nominal Dk/t**	45.4 × 10^−9^

**Notes:**

* Dk: (cm^2^ * mL O_2_)/(s * mL * hPa).

** Dk/t: (cm * mL O_2_)/(s * mL * hPa).

Mean ± SD (maximum and minimum) value are summarized. BOZD, back optic zone diameter; BOZR, back optic zone radius.

After lens insertion, the subjects waited 10 to 15 min to reduce the lacrimal reflex and were seated at a slit-lamp (SL-D2; Topcon, Japan) to be explored with blue light and a Boston yellow Kodak Wratten filter. Later, a drop of sterile saline solution was applied to a fluorescein strip, and excess fluid was shaken off before applying the strip flat to the superior bulbar conjunctiva in both eyes (Gasson & Morris, 2015). Subjects were advised to blink three or four times, and photographs of the fluorescein pattern under the contact lens in each eye were collected approximately 30 s after fluorescein instillation (Wolffsohn, Tharoo & Lakhlani, 2015) immediately after opening the eyes (when was necessary lids were separated and/or lenses were centered to guarantee good quality picture). Poor quality pictures, defocused pictures or pictures with decentered lenses were discarded. In all cases, JPEG image compression was applied.

After the photographs of fluorescein patterns from both eyes were captured -no more than 30 min of lens wear-, lenses were removed, and corneal integrity was assessed to ensure subjects’ ocular surface health.

### Algorithm development and image processing

**Figure 1:**
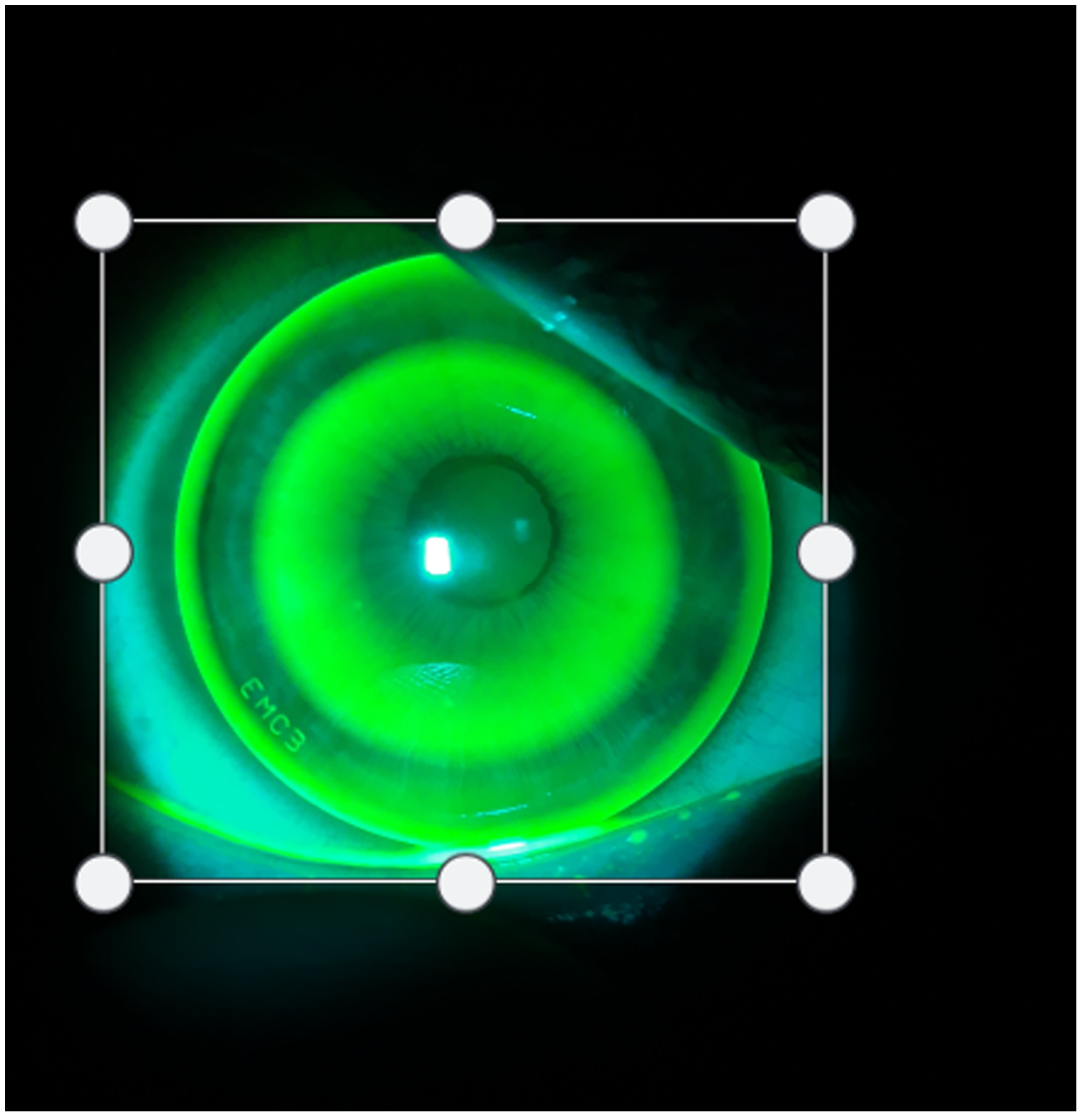
Fluorescein pattern cropping.

Fluorescein pattern photographs were exported directly from the capture software in JPG format. Each image was manually cropped to delete information unrelated to the fluorescein pattern and guarantee that the fluorescein pattern was centered in the picture. For this task, we used the software Paint 3D (Microsoft), and the images were saved with JPEG image compression (Fig. 1).

**Figure 2:**
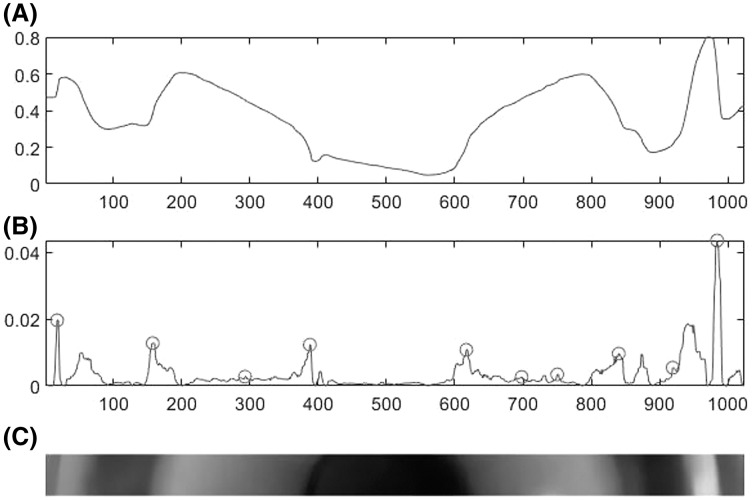
Summary of image processing. (A) Intensity profile of the fluorescein pattern chosen for analysis. (B) Absolute value of the first derivative of the intensity profile and the local maxima (note that 10 peaks are detected but just eight correspond with fluorescein image edges). (C) Horizontal meridian (60 × 1,000 pixels) of the fluorescein pattern chosen for analysis. X axes represents horizontal image pixels and Y axis image intensity ratio.

The images were processed with a customized algorithm written in the MATLAB programming environment (MATLAB R2019b for Windows; Intel® Core™ i7-7700 CPU @ 3.6 GHz). The algorithm combines a set of operations that have been previously used in other automated eye image analysis methods (Gonzalez, Woods & Eddins, 2010) (Fig. 2): (1) each image was proportionally resized to a width of 1,000 pixels; (2) the red and blue components from the red-green-blue (RGB) color space were removed to exclusively keep the green component since it shows the most relevant information; (3) the image contrast was maximized; (4) a width of 60 pixel of the centre horizontal meridian was chosen for analysis since the fluorescein horizontal meridian intensity is generally symmetrical (Wolffsohn, van der Worp & de Brabander, 2013); (5) the intensity profile of the image was plotted; and (6) finally, the absolute value of the first derivative of the intensity profile was calculated, and the local maxima (peaks) were selected to detect the image edges of each fluorescein zone. After image processing, two independent observers measured all fluorescein zones. The measures in pixels were converted to millimeters using the value of the total lens diameter (provided by the manufacturer).

**Figure 3:**
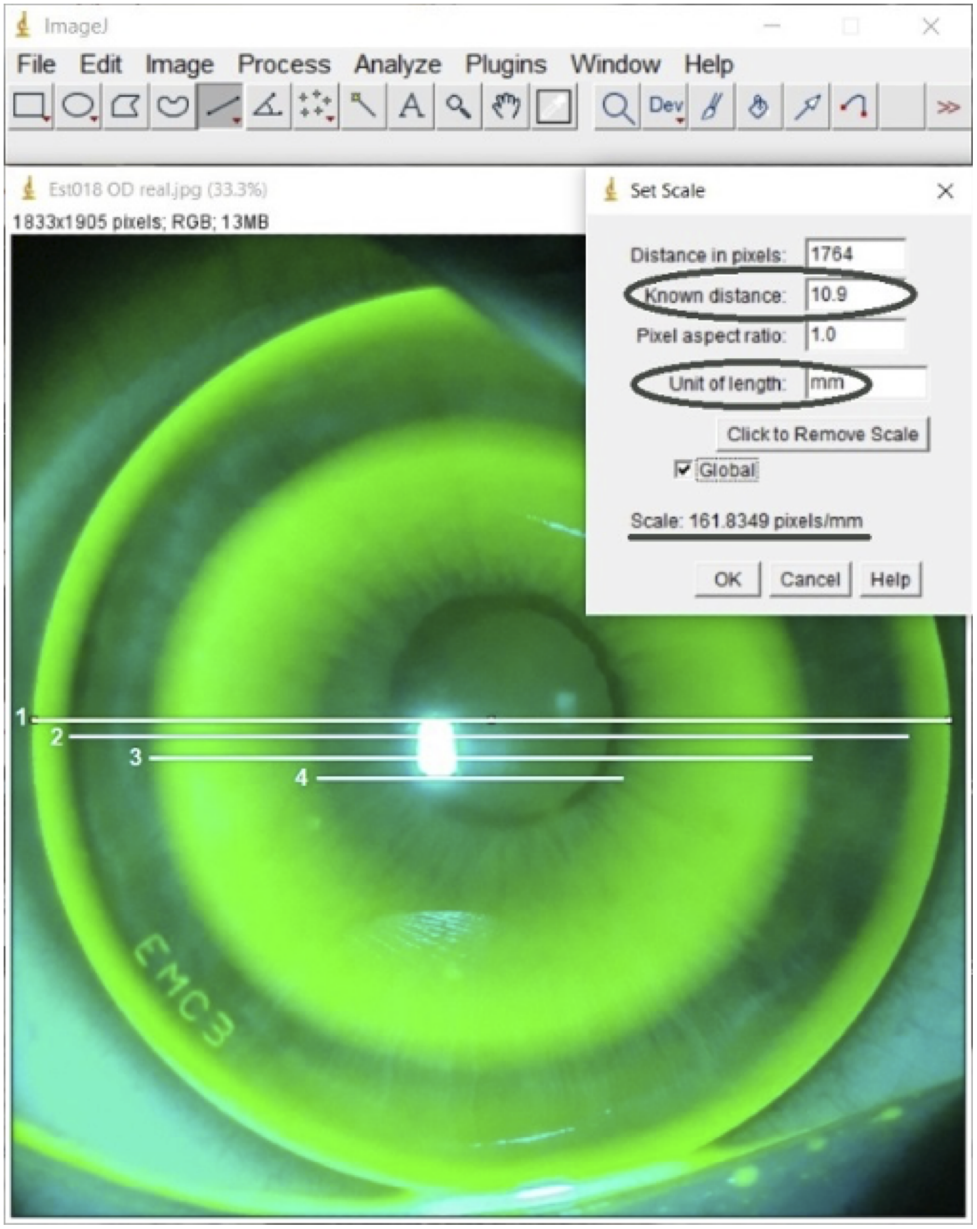
ImageJ screenshot of measurement procedure conducted by each observer. All measurements were conducted at centre of the image (horizontal meridian) but lines represent how each fluorescein zone wide was calculated; total diameter width [1], edge lift zone width [(1−2)/2]; mid-peripheral bearing width [(2−3)/2]; tear reservoir width [(3−4)/2] and central bearing width (4).

Additionally, the fluorescein pattern zones were measured by the two independent observers using the measurement calipers tool (Fig. 3) of the National Institute of Health open-access software ImageJ (version 1.46r, USA) (Rasband, 1997). ImageJ is an open-source image software focused on biological image analysis that allows for observer-dependent measurements. The total lens diameter was used to convert pixels to millimeters.

### Data analysis

Statistical analysis was performed using the SPSS 24.0 (SPSS, Chicago, IL, USA) statistical package for a Mac. The deviations of the variables from a normal distribution were assessed using the Kolmogorov-Smirnov test (*P* > 0.05 indicated that the data were normally distributed). Descriptive data analysis was performed using the mean ± standard deviation (SD).

We investigated the inter-observer agreement between the fluorescein pattern analysis conducted by two different observers that measured the width of the four main zones of the ortho-k fluorescein pattern, namely, the central bearing, tear reservoir, mid-peripheral bearing and edge lift zones, with the newly developed semiautomatic MATLAB algorithm and with ImageJ. Differences between both observers’ measurements were determined with a paired Student’s t-test (*P* values < 0.05 were considered significant) and Pearson correlation coefficient were also calculated.

Graphs of the differences between the pairs of measurements obtained by each observer divided by the average of the means of each pair of readings were plotted, and the limits of agreement were calculated (mean of the difference ± 1.96 * SD), as suggested by Bland and Altman. (Bland & Altman, 1986) Exact 95% confidence intervals for the repeatability limits of agreement were also calculated and plotted (Carkeet, 2015).

Also, agreement between ImageJ and the newly developed semiautomatic MATLAB algorithm results for the measurement of each fluorescein pattern zone was assessed with a paired Student’s t-test (*P* values < 0.05 were considered significant), and the limits of agreement and Pearson correlation coefficient were calculated.

## Results

Fluorescein patterns were measured in the photographs of all participants (26 eyes) using the new semiautomatic algorithm and ImageJ software. No subjects had significant biomicroscopic signs (grade > 1, Efron grading scale) of contact lens complications.

**Figure 4:**
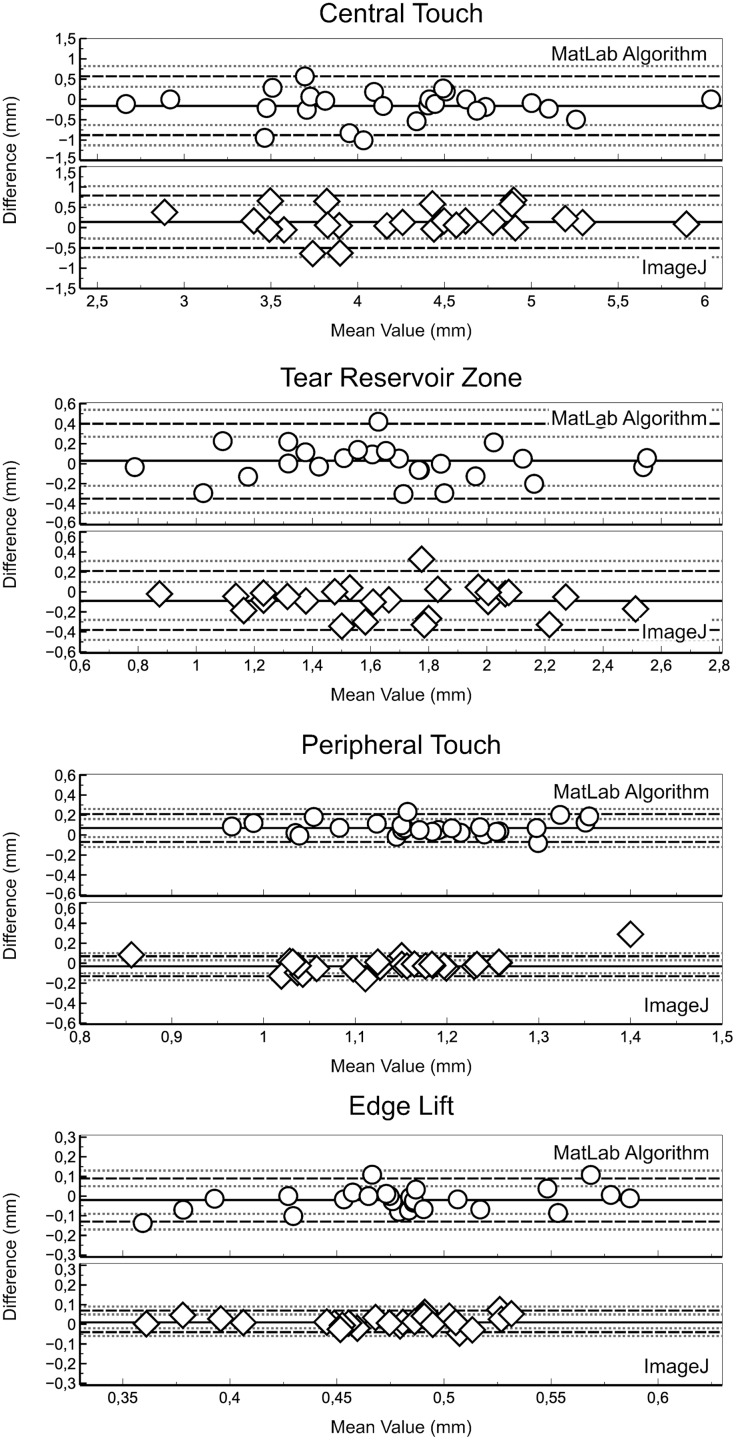
Bland-Altman plot showing the agreement between the fluorescein zone widths measured by both observer with each software (new semiautomatic MATLAB algorithm and ImageJ). Black solid lines showed mean difference and dotted lines LoA. Grey dotted lines showed 95% CI of LoA.

Good agreement between inter-observer measurements was also achieved for all fluorescein pattern zones measured with the new semiautomatic algorithm and ImageJ (Fig. 4). Regarding the measures achieved with the new semiautomatic algorithm central touch showed a mean difference of −0.16 ± 0.37 with LoA (95% CI) ranging from 0.57 [0.31 to 0.83] to −0.88 [−1.14 to −0.62] (*P* = 0.01), tear reservoir zone a mean difference of 0.03 ± 0.19 with LoA (95% CI) ranging from 0.4 [0.27 to 0.53] to −0.35 [−0.48 to −0.22] (*P* = 0.03), peripheral touch a mean difference of 0.07 ± 0.07 with LoA (95% CI) ranging from 0.21 [0.16 to 0.26] to −0.07 [−0.12 to −0.02] (*P* = 0.13) and edge lift a mean difference of −0.02 ± 0.06 with LoA (95% CI) ranging from 0.09 [0.05 to 0.13] to −0.13 [−0.17 to −0.09] (*P* = 0.15). Similar agreement was found regarding ImageJ measures, where central touch showed a mean difference of 0.14 ± 0.33 with LoA (95% CI) ranging from 0.79 [0.56 to 1.02] to −0.5 [−0.73 to −0.27] (*P* = 0.03), tear reservoir zone a mean difference of −0.09 ± 0.15 with LoA (95% CI) ranging from 0.21 [0.11 to 0.31] to −0.38 [−0.48 to −0.28] (*P* < 0.01), peripheral touch mean difference of −0.03 ± 0.05 with LoA (95% CI) ranging from 0.07 [0.04 to 0.1] to −0.13 [−0.16 to −0.1] (*P* = 0.01) and edge lift a mean difference of 0.01 ± 0.03 with LoA (95% CI) ranging from 0.07 [0.05 to 0.09] to −0.04 [−0.06 to −0.02] (*P* = 0.04). However, small statistically significant differences between software programs in some fluorescein pattern zones (Table 2) have found, that could have limited impact in clinical practice (not clinically significant).

**Table 2:** Summary of each fluorescein zone measured by each observer and software (new semiautomatic MATLAB algorithm and ImageJ).

	Program	Observer #1 M ± SD (95% CI)	Observer #2 M ± SD (95% CI)	Difference M ± SD (LoA)	*P*
Central bearing	MATLAB	4.12 ± 0.76 [3.82–4.43]	4.28 ± 0.76 [3.97–4.59]	−0.16 ± 0.34 [−0.88 to 0.57] r = 0.88 (*P* < 0.01)	0.04 (T_25_ = −2.15)
	ImageJ	4.38 ± 0.72 [4.09–4.68]	4.16 ± 0.77 [3.85–4.46]	0.14 ± 0.33 [−0.50 to 0.79] r = 0.89 *P* < 0.01	0.04 (T_25_ = 2.18)
	P′	0.02 (T_25_ = −2.50)	0.01 (T_25_ = 2.79)	–	–
Tear reservoir zone	MATLAB	1.70 ± 0.47 [1.51–1.89]	1.67 ± 0.45 [1.49–1.85]	0.03 ± 0.19 [−0.35 to 0.40] r = 0.91 *P* < 0.01	0.51 (T_25_ = 0.66)
	ImageJ	1.63 ± 0.40 [1.47–1.79]	1.76 ± 0.45 [1.56–1.94]	−0.09 ± 0.15 [−0.38 to 0.21] r = 0.82 *P* < 0.01	0.02 (T_25_ = −2.48)
	P′	0.21 (T_25_ = 1.29)	<0.01 (T_25_ = −3.94)	–	–
Mid-peripheral bearing	MATLAB	1.21 ± 0.11 [1.17–1.26]	1.14 ± 0.11 [1.10–1.19]	0.07 ± 0.07 [−0.07 to 0.21] r = 0.80 *P* < 0.01	<0.01 (T_25_ = 5.20)
	ImageJ	1.12 ± 0.13 [1.08–1.18]	1.14 ± 0.10 [1.10–1.18]	−0.03 ± 0.05 [−0.13 to 0.07] r = 0.83 *P* < 0.01	0.32 (T_25_ = −0.93)
	P′	<0.01 (T_25_ = 4.19)	0.93 (T_25_ = −0.08)	–	–
Edge lift	MATLAB	0.47 ± 0.07 [0.44–0.50]	0.49 ± 0.05 [0.47–0.51]	−0.02 ± 0.06 [−0.13 to 0.09] r = 0.62 *P* < 0.01	0.09 (T_25_ = −1.74)
	ImageJ	0.49 ± 0.10 [0.45–0.53]	0.46 ± 0.05 [0.44–0.48]	0.01 ± 0.03 [−0.04 to 0.07] r = 0.82 *P* < 0.01	0.10 (T_25_ = 1.69)
	P′	0.31 (T_25_ = −1.04)	<0.01 (T_25_ = 4.24)	–	–

**Note:**

LoA, Limits of agreement. Paired t Test value is represented to show differences between observers (P) and between MATLAB and ImageJ (P′).

**Figure 5:**
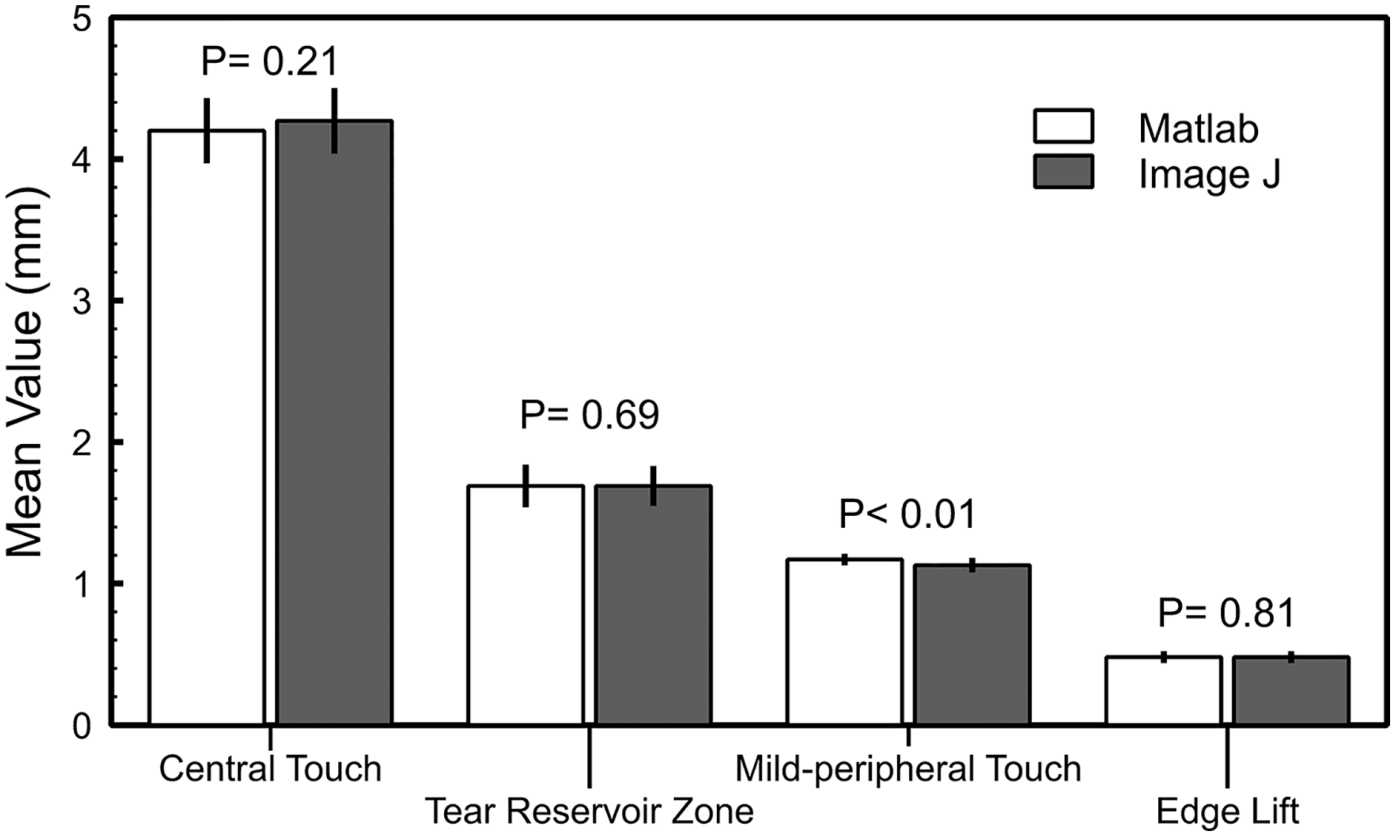
Summary of the measurement of the central bearing, tear reservoir zone, mid-peripheral bearing and edge lift of the fluorescein pattern with the new semiautomatic MATLAB algorithm and ImageJ software. Error bars represent CI 95%.

The new semiautomatic algorithm and ImageJ software provide close measurements of the central bearing, tear reservoir zone, mid-peripheral bearing and edge lift fluorescein pattern widths (Fig. 5) with good agreement (Table 3), lower than 0.07 mm. Statistically significant differences suggest that measurements achieved with both software programs and observers could be not interchangeable (Tables 2 and 3), but could be not clinically significant, due the small difference found (lower than 0.16 mm).

**Table 3:** Agreement between both software programs (new semiautomatic MATLAB algorithm and ImageJ) of each fluorescein pattern zone measurement.

	MATLAB M ± SD (95% CI)	ImageJ M ± SD (95%CI)	Difference M ± SD (LoA)	*P*
Central bearing	4.20 ± 0.74 [3.99–4.41]	4.27 ± 0.69 [4.06–4.48]	−0.07 ± 0.28 [−0.62 to 0.48] r = 0.93 (*P* < 0.01)	0.21 (T_25_ = −1.27)
Tear reservoir zone	1.69 ± 0.41 [1.56–1.81]	1.69 ± 0.45 [1.57–1.81]	−0.01 ± 0.14 [−0.28 to 0.26] r = 0.95 *P* < 0.01	0.69 (T_25_ = −0.41)
Mid-peripheral bearing	1.17 ± 0.11 [1.15–1.21]	1.13 ± 0.10 [1.10–1.17]	0.05 ± 0.05 [−0.05 to 0.15] r = 0.86 *P* < 0.01	<0.01 (T_25_ = 3.81)
Edge lift	0.48 ± 0.06 [0.46–0.50]	0.47 ± 0.06 [0.46–0.50]	0.01 ± 0.04 [−0.06 to 0.08] r = 0.44 *P* = 0.02	0.81 (T_25_ = 0.24)

**Note:**

LoA, Limits of agreement.

## Discussion

This article presents a new semiautomatic algorithm to detect the edges of the main zones of ortho-k fluorescein patterns in order to conduct a quantitative analysis of the width of the central bearing, tear reservoir zone, mid-peripheral bearing and edge lift areas along the horizontal meridian. Few reports have explored the use of image processing procedures to automatically identify base curves in RGP lenses (Hashemi et al., 2020) or ortho-k lenses, (Zhang et al., 2017) but none allow the measurement of the fluorescein pattern zones.

In fact, these previous reports usually need to use a specific corneal tomography device (Pentacam) that is not available in all eye clinics who offer ortho-k. Although different computer-aided ortho-k lens fitting systems are currently available, contact lens practitioners must conduct a highly subjective and practitioner-dependent (Wolffsohn, van der Worp & de Brabander, 2013) analysis of the fluorescein pattern prior to dispensing the ortho-k lenses for the first overnight wear (Kim et al., 2018). For this reason, fluorescein pattern assessment does not allow the identification of the correct ortho-k lens outcome (Mountford, Cho & Chui, 2005) until after the first overnight wear topographic effect assessment was conducted. Previous reports suggest that static fluorescein analysis is insufficient for lens fitting assessments, mainly due to the slight movement of the reverse geometry lenses, the close distance between the lens and cornea (Mountford, Cho & Chui, 2005; Orsborn et al., 1989) and the practitioner dependency (Vincent et al., 2021; Wolffsohn, van der Worp & de Brabander, 2013). However, if quantitative fluorescein pattern analysis could be conducted, reducing the subjective dependency of this analysis could be possible, and prior to first overnight wear, ortho-k lenses assessment could help practitioners to define optimal lens parameters. Therefore, it is necessary to improve fluorescein pattern analysis in ortho-k practice to reduce inter-examiner differences and dependence (Wolffsohn, van der Worp & de Brabander, 2013).

Edge detection of each ortho-k fluorescein pattern zone is an indispensable step to acquire numerical parameters that are relevant in terms of their practical use in clinical and research practice. Therefore, the accuracy of the algorithm responsible for this process is an extremely important issue, but to the best of our knowledge, no previous report has addressed this issue. We used a custom MATLAB algorithm to process standard fluorescein pattern images, detect the four main fluorescein pattern zones along the horizontal meridian, and provide a measurement of their widths with small differences between observers and manual (ImageJ) measurement that could have limited clinical relevance (Ranganathan, Pramesh & Buyse, 2015) –although some measurements showed statistically significant differences–. This approach showed good inter-subject agreement, with close measurements lower than 0.16 mm, suggesting that these values could reduce eye care practitioner dependency and subjectivity in ortho-k fluorescein pattern analysis (Vincent et al., 2021; Wolffsohn, van der Worp & de Brabander, 2013). However, further research will be neccesary to clarify the impact of these differences in ortho-k practice and myopia control.

The new algorithm provides a central bearing width (between 3.99 and 4.41 mm, Tables 2 and 3) close to the previously recommended range (3.5 to 4.5 mm) in ortho-k practice (Gasson & Morris, 2015). The fluorescein central bearing diameter must be lower than the back optic zone diameter (BOZD) (D0 = 6.79 ± 0.38 mm, Table 1) because the distance between the lens and cornea is thinner in apex (lower than 15–20 microns in the central corneal area (Wolffsohn, van der Worp & de Brabander, 2013)) but increases toward the corneal periphery, so the tear layer thickness under the area close to the edge of the BOZD (D0) and the junction with a reverse curve zone (D1) allows fluorescein clearance. In fact, Kim et al. (2018) found a significant epithelium and stroma thickening in the mid-peripheral corneal zone (5 to 6 mm) that nearly coincides with the reverse curve zone of the fluorescein pattern measured (5.74 to 6.04 mm -95% IC of the sum of central bearing and tear reservoir zone widths-), suggesting that fluorescein pattern measurements can be correlated with topographic outcomes. Because tear film pooling zones are dependent on the lens design, it should be difficult to conduct topographic change comparisons achieved with different contact lens designs, but future fluorescein pattern measurements can allow the study of different lens design impacts in ortho-k practice. However, this issue requires further research comparing fluorescein pattern measurements and topographic changes after ortho-k lens wear.

Regarding the mid-peripheral bearing measurement, a slightly wider width (1.14 to 1.21 mm) than the expected width of the designed alignment width (D2) (0.85 mm) was found because cornea flattens towards the periphery. Finally, an edge lift width (D3) of 0.6 mm nearly coincides with the edge lift width (0.46 to 0.50 mm) measured in the fluorescein pattern. The differences between the designed alignment zone and edge lift could be related to topographic differences in the peripheral lens fitting that allow different tear film layer thicknesses under the lens but suggest that new algorithm measurements are plausible with the reverse geometry lens design used in this study.

The results of this new algorithm conducted by two different observers found higher inter-subject agreements (Bland–Altman test and Pearson’s correlation coefficient shown in Tables 2 and 3), similar to the agreements achieved with the manual measurements achieved with common image analysis software (ImageJ), but requiring a simple procedure to obtain fluorescein pattern measurements compared with manual procedure necessary with ImageJ. This finding suggests that the new algorithm could be useful in clinical practice, reducing the dependency of practitioner experience and guaranteeing a semiautomatic diagnostic lens fitting analysis to provide valuable information for ortho-k lens adaptation (Zhang et al., 2017). However, further improvement in this proof of concept is required before to be introduced in clinical practice.

Relatively small changes in the lens parameters (whose determination requires the localization of the fluorescein pattern zone edge) may affect the accuracy of the ortho-k lens effect and its center (Maseedupally et al., 2016; Zhang et al., 2017). The newly proposed algorithm showed the precise measurement of each fluorescein pattern zone with great inter-subject and inter-software agreement, suggesting that their outcomes could be sufficiently precise to be used in clinical and/or in research practice. Obtaining the smallest possible measurement error is particularly important in this case because the fluorescein pattern is limited to the lens diameter (usually between 10.0 and 12.0 mm), and four different zones (diameters) must be assessed and measured in each case.

### Study limitations and future perspectives

Despite a small sample size, our results showed that the semiautomatic algorithm can provide measurements of each fluorescein pattern zone with limited examiner influence. The main objective of this study was to compare the new algorithm with standard, commonly used software (ImageJ), not conduct a clinical validation of this algorithm (this requires further research to define future clinical standards of each zone widths and others applications). Future improvements in the developed algorithm are necessary: first to allow automatic edge detection and reduce examiner influence in measurements; second to measure different corneal meridians, (for example, to provide the area and not just the horizontal width of the fluorescein pattern zone, which could be of interest if toric lenses are necessary), (Maseedupally et al., 2016) third to assess fluorescein measurement after over-night wear of ortho-k lenses and finally simplifying its use, automating the treatment of images and the saving of the results. Moreover, automatic fluorescein pattern measurements will improve data collection in single and multicenter prospective clinical research projects to determine changes in fluorescein patterns with time and treatment, correlate fluorescein changes with topographical changes (treatment zone), or assess the impact of lens design or manufacturer on the clinical efficacy in myopia control with ortho-k.

In summary, the proposed algorithm can expedite ortho-k practice by reducing the time and practitioner dependency in fluorescein assessment, simplifying the method of recording the ortho-k lens fit, facilitating communication in large practices with multiple eye-care specialists, colleagues, lens manufacturers or consultants (Wolffsohn, van der Worp & de Brabander, 2013). Additionally, this algorithm could be used to prescribe lenses for the first overnight use, to reduce practitioner dependency, could allow the collection of data that could be used in large clinical trials to improve the identification of key clinical factors in lens centration or ortho-k effects, or perform the classification or verification of patients, introduce artificial intelligence networks or deep learning-based methods to predict ortho-k effects, and help clinicians manage myopia progression to provide better care and follow-up assessing or monitoring fluorescein pattern in ortho-k. Thus, this algorithm could be implemented in commercially available tools, such as reverse geometry fitting software or other.

## Conclusions

The use of reverse geometry RGP lenses has opened new possibilities in the field of myopia correction and control. In this study, the authors addressed a basic problem related to the subjective analysis of the fluorescein pattern in ortho-k practice, by proposing a new algorithm for the semiautomatic analysis and measurement of fluorescein patterns. In this method, the edges of its four different zones are detected, providing high inter-subject repeatable measurements, which open the possibility of new clinical and research options in ortho-k practice.

## Supplemental Information

10.7717/peerj.14068/supp-1 Supplemental Information 1 Dataset of study data. Measurements collected by researchers using custom algorithm and ImageJ software of fluerescein pattern.

## Additional Information and Declarations

### Data Availability

The following information was supplied regarding data availability: The raw measurements are available in the Supplemental Files.
